# Supplementary material for: Genomic heterogeneity of NAD(P)H dehydrogenase predisposes Cryptosporidium to clofazimine resistance
Source: Nat Microbiol. 2026 May 13;11(6):1559–72. doi: 10.1038/s41564-026-02331-5 (PMC13236581; doi:10.1038/s41564-026-02331-5)
Supplement: Supplementary file 1 — Reporting Summary [file 41564_2026_2331_MOESM1_ESM.pdf]

nature portfolio

Corresponding author(s): Striepen Boris

Last updated by author(s): Jan 26, 2026

Reporting Summary

Nature Portfolio wishes to improve the reproducibility of the work that we publish. This form provides structure for consistency and transparency in reporting. For further information on Nature Portfolio policies, see our Editorial Policies and the Editorial Policy Checklist.

Statistics

For all statistical analyses, confirm that the following items are present in the figure legend, table legend, main text, or Methods section.

n/a	Confirmed	
☐	☒	The exact sample size (*n*) for each experimental group/condition, given as a discrete number and unit of measurement
☐	☒	A statement on whether measurements were taken from distinct samples or whether the same sample was measured repeatedly
☐	☒	The statistical test(s) used AND whether they are one- or two-sided<br>*Only common tests should be described solely by name; describe more complex techniques in the Methods section.*
☒	☐	A description of all covariates tested
☒	☐	A description of any assumptions or corrections, such as tests of normality and adjustment for multiple comparisons
☐	☒	A full description of the statistical parameters including central tendency (e.g. means) or other basic estimates (e.g. regression coefficient) AND variation (e.g. standard deviation) or associated estimates of uncertainty (e.g. confidence intervals)
☐	☒	For null hypothesis testing, the test statistic (e.g. *F*, *t*, *r*) with confidence intervals, effect sizes, degrees of freedom and *P* value noted<br>*Give P values as exact values whenever suitable.*
☒	☐	For Bayesian analysis, information on the choice of priors and Markov chain Monte Carlo settings
☒	☐	For hierarchical and complex designs, identification of the appropriate level for tests and full reporting of outcomes
☒	☐	Estimates of effect sizes (e.g. Cohen's *d*, Pearson's *r*), indicating how they were calculated

Our web collection on statistics for biologists contains articles on many of the points above.

Software and code

Policy information about availability of computer code

Data collection	A Leica DM6000B Upright Widefield Fluorescence Microscope was used for timecourse and infected mouse enterocyte imaging with Leica Application Suite X version 3.7.4.23463 software.<br>A GE DeltaVision OMX Structured Illumination Super-Resolution Microscope was used for high resolution imaging of parasites with Acquire SR Acquisition control version 4.5.10296-1 software.<br>A Leica STELLARIS 8 FALCON confocal microscope with Leica LAS X software version 4.6.1.27508 was used for imaging samples prepared by ultrastructure expansion microscopy. Samples were processed using the Leica Lightning deconvolution and analyzed with the Fiji software.
Data analysis	Imaging analysis:<br>Fiji v2.26.0/1.54n<br><br>Computation analysis:<br>Bulk Segregant Analysis – BWA v0.7.17, SAMTools v1.19.2, GATk v4.6.2, magrittr v2.0.3, QTLseqr v0.7.0, ggplot2 v4.0.0, dplyr v1.1.4; R v4.42, RStudio-Server v2025.05.0-496, Automated_Bulk_Segregant_Analysis v0.1<br>Amplicon-seq Analysis - BWA v0.7.17, SAMTools v1.19.2, GATk v4.6.2, IGV v2.19.5<br>Global NDH2 Allele Frequency Analysis – sra2vcf v0.1, Python v3.10.11, SRA Toolkit v3.0.5, TrimGalore v0.6.10, BWA v0.7.17,minimap2 v2.26, STAR v2.7.10b, SAMTools v1.19.2, BCFTools v1.22, R v4.42, MultiQC v 1.25.2, RStudio-Server v2025.05.0-496<br>Global Essential_Nonessential Gene Analysis – SnpEff v5.2e, SnpSift v5.2e, R v4.42, RStudio-Server v2025.05.0-496<br>All code is available with MIT free license through Github repositories sra2vcf(https://github.com/ruicatxiao/sra2vcf) and cparvum_ndh2_clofazimine-resistance (https://github.com/ruicatxiao/cparvum_ndh2_clofazimine-resistance)

For manuscripts utilizing custom algorithms or software that are central to the research but not yet described in published literature, software must be made available to editors and reviewers. We strongly encourage code deposition in a community repository (e.g. GitHub). See the Nature Portfolio guidelines for submitting code & software for further information.

Data

Policy information about availability of data

All manuscripts must include a data availability statement. This statement should provide the following information, where applicable:

- Accession codes, unique identifiers, or web links for publicly available datasets
- A description of any restrictions on data availability
- For clinical datasets or third party data, please ensure that the statement adheres to our policy

Whole genome raw sequencing data and the raw amplicon sequencing data have been deposited in the NCBI's Sequencing Read Archive database under Bioproject numbers PRJNA1336748 and PRJNA1337473, respectively.

Research involving human participants, their data, or biological material

Policy information about studies with human participants or human data. See also policy information about sex, gender (identity/presentation), and sexual orientation and race, ethnicity and racism.

Reporting on sex and gender	N/A
Reporting on race, ethnicity, or other socially relevant groupings	N/A
Population characteristics	N/A
Recruitment	N/A
Ethics oversight	N/A

Note that full information on the approval of the study protocol must also be provided in the manuscript.

Field-specific reporting

Please select the one below that is the best fit for your research. If you are not sure, read the appropriate sections before making your selection.

☒ Life sciences ☐ Behavioural & social sciences ☐ Ecological, evolutionary & environmental sciences

For a reference copy of the document with all sections, see nature.com/documents/nr-reporting-summary-flat.pdf

Life sciences study design

All studies must disclose on these points even when the disclosure is negative.

Sample size	Mouse experiments to isolate transgenic parasites were conducted with 4-5 mice (following Vinayak et al, Nature 523 : 477-480) and measurements of oocyst shedding used groups of 3 mice (Manjunatha et al, Nature 546: 376-380 and Shaw et al, PNAS 121: e2313210120). For microscopy-based experiments to measure HA expression we used one biological replicate and counted 10-11 randomly chosen fields per condition. n = 439 – 638 individual cells were used for the analysis. IC50s were conducted in at least 2 biological replicates, each with 5 technical replicates (Vinayak et al, Nature 523 : 477-480).
Data exclusions	No data were excluded
Replication	All attempts at replication were successful, with all IC50s repeated 2 times, except the IC50 of the non selected and selected cross (Fig 1e).
Randomization	Mice were initially chosen at random, but sex and age-matched between treatment and control group. Other experiments did not lend themselves to randomization due to small number of variations (e.g. plus or minus small molecule).
Blinding	Blinding was not used because it was not practical for a single central experimenter. Also in many experiments results were so clear that they would have subverted blinding

Reporting for specific materials, systems and methods

We require information from authors about some types of materials, experimental systems and methods used in many studies. Here, indicate whether each material, system or method listed is relevant to your study. If you are not sure if a list item applies to your research, read the appropriate section before selecting a response.

Materials & experimental systems

n/a	Involved in the study
☐	☒ Antibodies
☐	☒ Eukaryotic cell lines
☒	☐ Palaeontology and archaeology
☐	☒ Animals and other organisms
☒	☐ Clinical data
☒	☐ Dual use research of concern
☒	☐ Plants

Methods

n/a	Involved in the study
☒	☐ ChIP-seq
☐	☒ Flow cytometry
☒	☐ MRI-based neuroimaging

Antibodies

Antibodies used

Primary Antibodies:
1 - Rat anti-HA High Affinity,from rat IgG1 (Roche, cat. 11867423001, Clone #3F10) (IFA), 1:1000 dilution
2 - Anti-MBP-IMC3 fusion protein expressed in BL21 E. coli, from rabbit antisera (IFA), 1:500 dilution
3 - Vicia Villosa Lectin (VVL, VVA), Biotinylated (Vector Laboratories, cat. B-1235, Lot #ZD0509) (IFA), 1:1000 dilution
4- HA-Tag Rabbit mAB (Cell Siganling Technology, REF 3724S, Clone #C29F4) (Western blot), 1:1000 dilution,
5- H3pan Antibody (Diagenode, REF C15200011, Lot #003) (Western blot), 1:1000 dilution,

Secondary antibodies:
IRDye-800CW goat anti-rabbit (REF 926-32211, LOT #D00804-07) (Western blot), 1:10000 dilution
IRDye-680RD goat ant-mouse (REF 926-68070, LOT #D00804-13) (Western blot), 1:10000 dilution
Goat anti-Rat IgG (H+L) Cross-Adsorbed Secondary Antibody, Alexa Fluor™ 488 (Invitrogen, cat. A-11006, Lot #2048174) (IFA), 1:1000 dilution
Goat anti-Rabbit IgG (H+L) Cross-Adsorbed Secondary Antibody, Alexa Fluor™ 594 (Invitrogen, cat. A-11012, Lot #2616076) (IFA), 1:1000 dilution
Streptavidin, Alexa Fluor™ 594 Conjugate (Invitrogen, cat. S11227, Lot #1872019) (IFA), 1:1000 dilution

Validation

1-HA has been used extensively in our lab: Tandel, J. et al. Life cycle progression and sexual development of the apicomplexan parasite Cryptosporidium parvum. Nat Microbiol 4, 2226-2236, doi:10.1038/s41564-019-0539-x (2019); Guerin, A. et al. Cryptosporidium uses multiple distinct secretory organelles to interact with and modify its host cell. Cell Host Microbe 31, 650-664 e656, doi:10.1016/j.chom.2023.03.001 (2023).
2-validated in Gubbels et al. (2004), https://doi.org/10.1016/j.molbiopara.2004.05.007)
3- VVL has been used extensively in the field to score Cryptosporidium: Sharling, L., et al. A Screening Pipeline for Antiparasitic Agents Targeting Cryptosporidium Inosine Monophosphate Dehydrogenase. PLoS Negl Trop Dis 4(8): e794, doi: 10.1371/journal.pntd.0000794 (2010).
4-HA has been used in our lab: Guerin, A. et al.
Cryptosporidium uses multiple distinct secretory organelles to interact with and modify its host cell. Cell Host Microbe 31, 650-664 e656, doi:10.1016/j.chom.2023.03.001 (2023)
5-Extensive validation data can be found here: https://www.diagenode.com/en/p/h3pan-monoclonal-antibody-classic-50-mg-100-ml

Eukaryotic cell lines

Policy information about cell lines and Sex and Gender in Research

Cell line source(s)	Human colorectal adenocarcinoma HCT-8 cells (ATCC: CCL-224TM)
Authentication	Cell lines not authenticated
Mycoplasma contamination	Cell lines were not tested for mycoplasma contamination
Commonly misidentified lines (See ICLAC register)	There are no commonly misidentified lines in this study

Animals and other research organisms

Policy information about studies involving animals; ARRIVE guidelines recommended for reporting animal research, and Sex and Gender in Research

Laboratory animals	IFN-gamma knockout mice (stock number: 002287) were purchased from Jackson Laboratory and maintained as a breeding colony at the University of Pennsylvania. Mice used for experiments ranged in age from 4 to 8 weeks. Mice were housed with a 12-hour dark/light cycle, a temperature between 65 – 73 degrees Fahrenheit, and a humidity level between 30 to 40 percent.
Wild animals	No wild animals were used in this study.
Reporting on sex	Both male and female mice were used to generate and propagate Cryptosporidium parvum strains and did not exhibit a difference in

Reporting on sex	oocyst shedding. Mice were sex and age matched for each experiment.
Field-collected samples	This study involves one C.parvum strain (KVI) which was collected in the field (Shaw et al., 2025; Cell Rep; https://doi.org/ 10.1016/ j.celrep.2025.116315)
Ethics oversight	All animal experimentation was approved by the Institutional Animal Care and Use Committee of the University of Pennsylvania (protocol #806292).

Note that full information on the approval of the study protocol must also be provided in the manuscript.

Plants

Seed stocks	N/A
Novel plant genotypes	N/A
Authentication	N/A

Flow Cytometry

Plots

Confirm that:

☒ The axis labels state the marker and fluorochrome used (e.g. CD4-FITC).

☒ The axis scales are clearly visible. Include numbers along axes only for bottom left plot of group (a 'group' is an analysis of identical markers).

☒ All plots are contour plots with outliers or pseudocolor plots.

☒ A numerical value for number of cells or percentage (with statistics) is provided.

Methodology

Sample preparation	Oocysts were purified from fecal material
Instrument	BD FACSymphony™ A3 | 5-Laser Cell Analyzer
Software	BD FacsDiva version 9.0
Cell population abundance	It is a purified sample. Mostly oocysts with some bacterial contamination.
Gating strategy	Oocysts were identified by size and plotted in a histogram for mNeonGreen expression intensity.

☒ Tick this box to confirm that a figure exemplifying the gating strategy is provided in the Supplementary Information.
